# Supplementary material for: Comprehensive Evaluation of End-Point Free Energy Techniques in Carboxylated-Pillar[6]arene Host–Guest Binding: III. Force-Field Comparison, Three-Trajectory Realization and Further Dielectric Augmentation
Source: Molecules. 2023 Mar 19;28(6):2767. doi: 10.3390/molecules28062767 (PMC10059726; doi:10.3390/molecules28062767)
Supplement: Supplementary file 1 [file molecules-28-02767-s001.zip › molecules-2276401-supplementary.pdf]

Supporting Information:

Comprehensive Evaluation of End-Point Free Energy Techniques in Carboxylated-Pillar[6]arene Host-guest Binding: III. Force-Field Comparison, Three-Trajectory Realization and Further Dielectric Augmentation

Xiao Liu[1], Lei Zheng[2,3], Chu Qin[1], Yalong Cong[4], John Z.H. Zhang[2,3,4,5], Zhaoxi Sun[6]

[1]*School of Mathematics, Physics and Statistics, Shanghai University of Engineering Science, Shanghai 201620, China*

[2]*NYU-ECNU Center for Computational Chemistry at NYU Shanghai, Shanghai 200062, China*

[3]*Department of Chemistry, New York University, NY, NY 10003, USA*

[4]*School of Chemistry and Molecular Engineering, East China Normal University, Shanghai, 200062, China*

[5]*Shenzhen Institute of Advanced Technology, Chinese Academy of Sciences, Shenzhen, Guangdong, China*

[6]*College of Chemistry and Molecular Engineering, Peking University, Beijing 100871, China*

Fig. S1. Torsional terms sharing exactly the same parameters under GAFF and GAFF2.

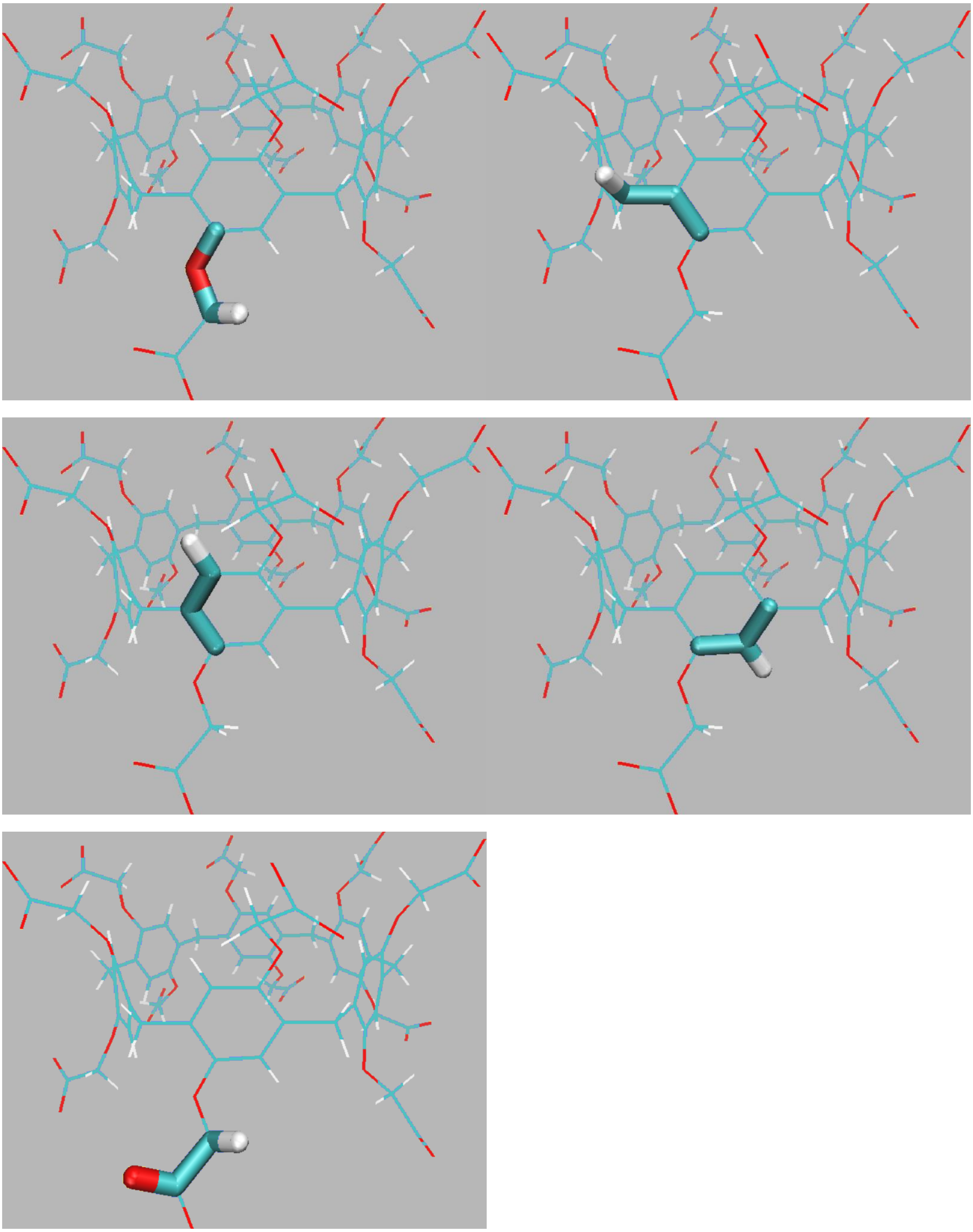

Fig. S2. Time series of the polar part of implicit-solvent contributions in host-guest, guest-only and host-only simulations with GAFF2 and TIP3P water. COM, LIG and REC in legends represent complex, ligand/guest and host/receptor, respectively. Snapshots in host-only simulations and the corresponding Conolly surfaces are presented to illustrate the structural feature of configurations with significantly different solvation and electrostatic energies.

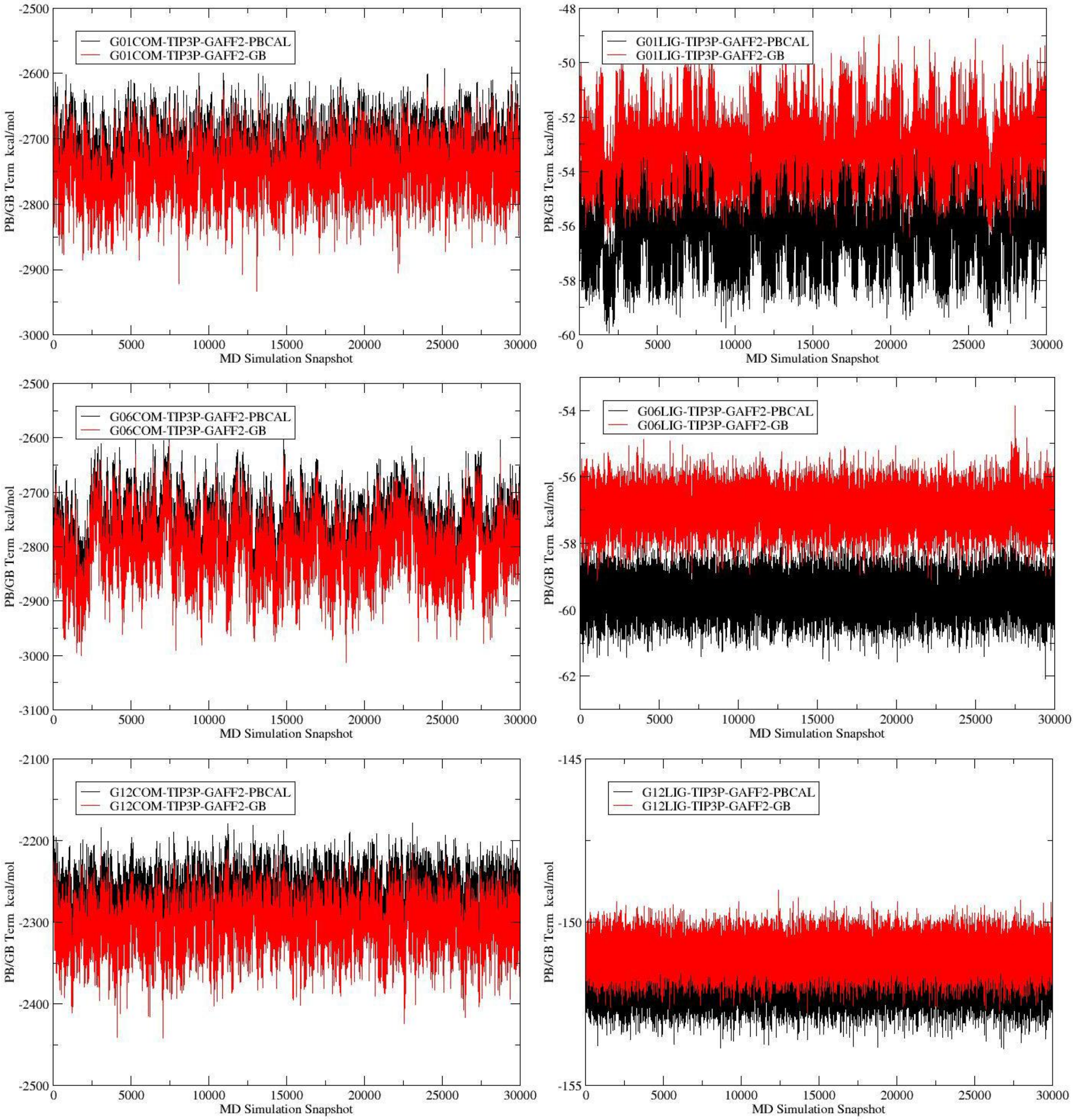

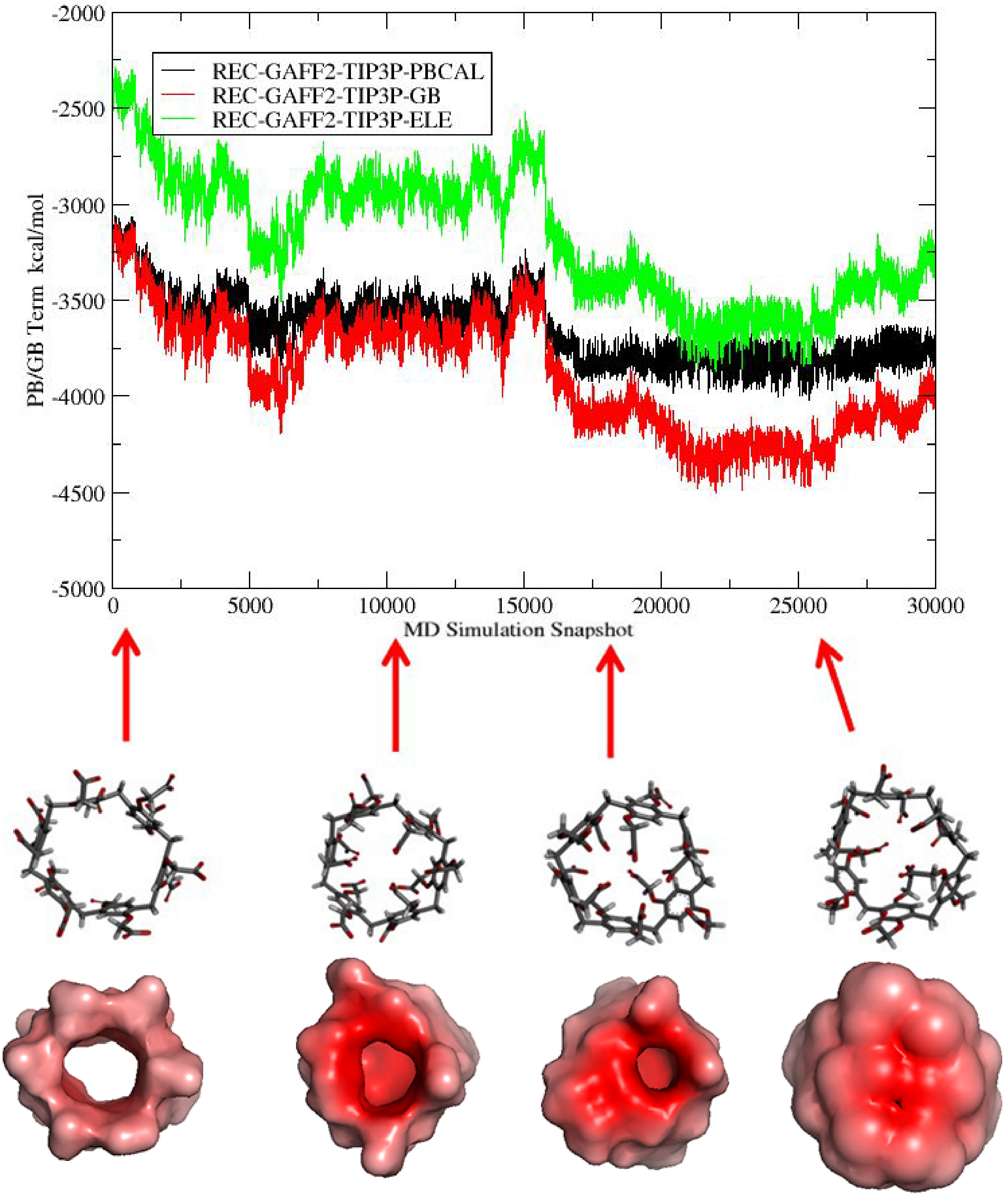
REC-GAFF2-TIP3P-PBCAL
REC-GAFF2-TIP3P-GB
REC-GAFF2-TIP3P-ELE
PB/GB Term kcal/mol
MD Simulation Snapshot
-2000
-2500
-3000
-3500
-4000
-4500
-5000
0
5000
10000
15000
20000
25000
30000

Fig. S3. Time series of the total polar contribution (polar solvation and electrostatics) in host-guest, guest-only and host-only simulations with GAFF2 and TIP3P water. COM, LIG and REC in legends represent complex, ligand/guest and host/receptor, respectively. PBELE and GBELE in the legend represents the total polar contribution in the enthalpy (i.e., PB/GB+ELE). Snapshots in host-only simulations are presented to illustrate the structural feature of configurations with significantly different polar energetics.

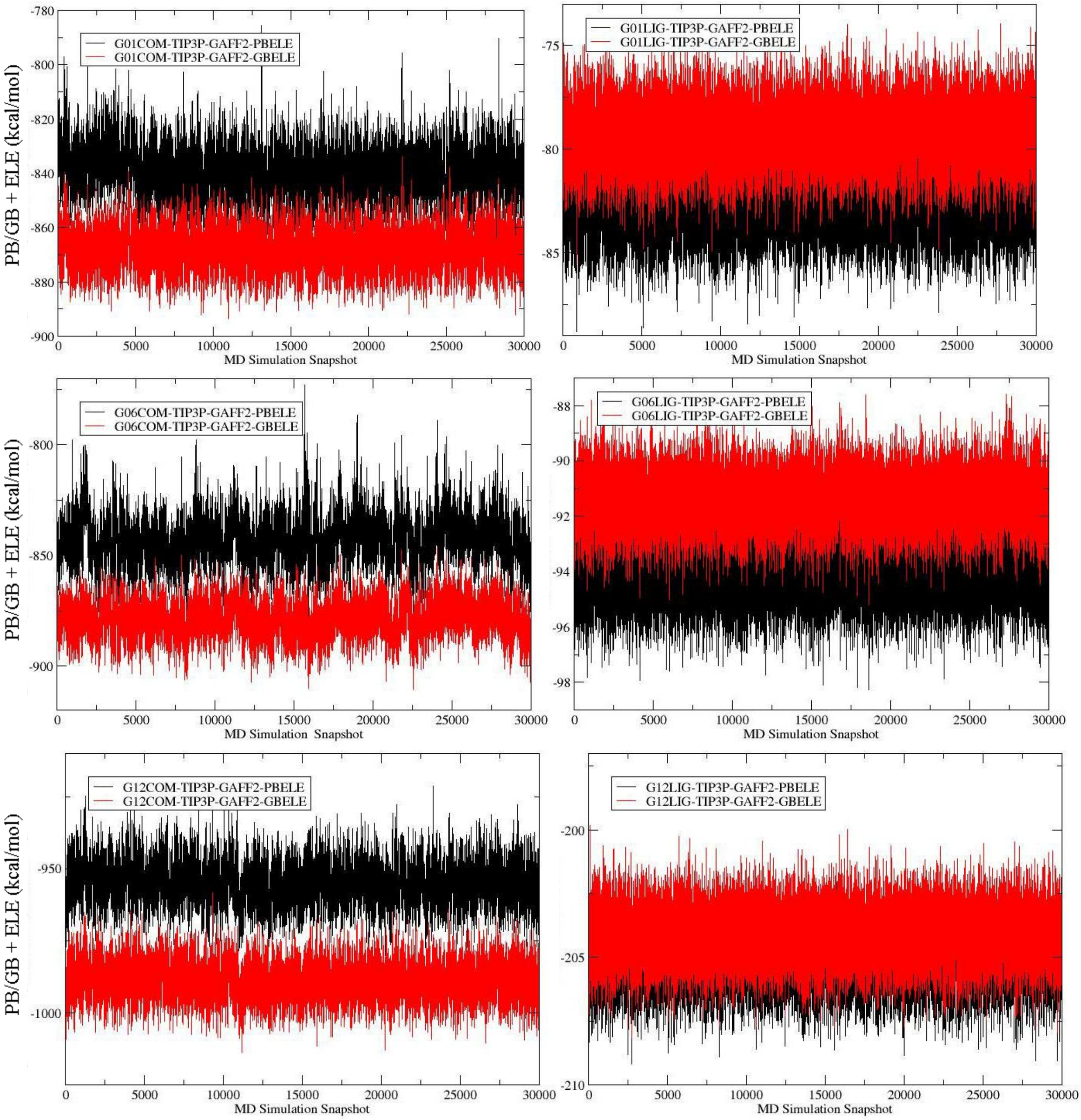

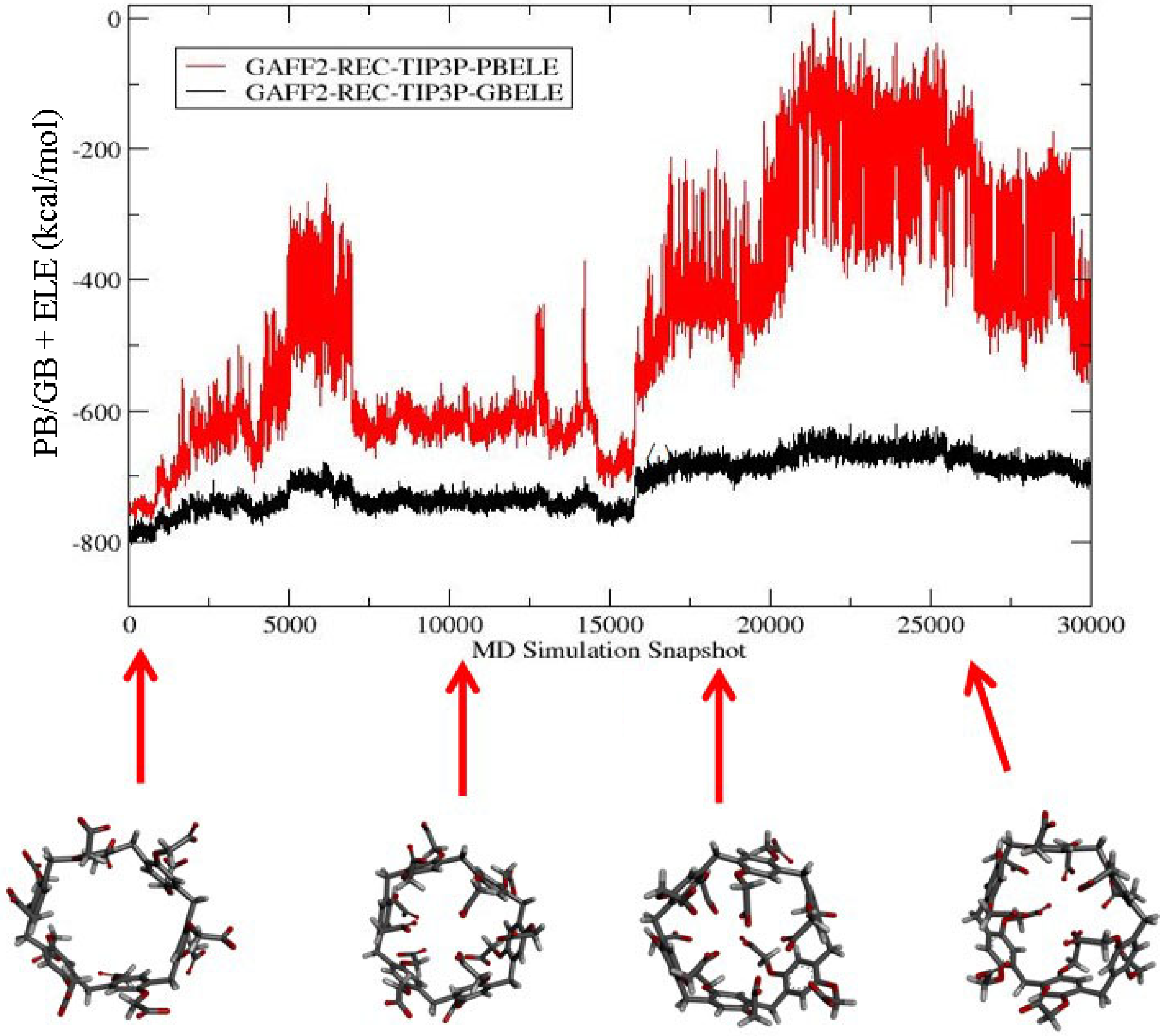
GAFF2-REC-TIP3P-PBELE
GAFF2-REC-TIP3P-GBELE
PB/GB + ELE (kcal/mol)
MD Simulation Snapshot
0
-200
-400
-600
-800
0
5000
10000
15000
20000
25000
30000

Fig. S4. Time series of the polar contribution in the host-only simulation under GAFF and TIP3P water.

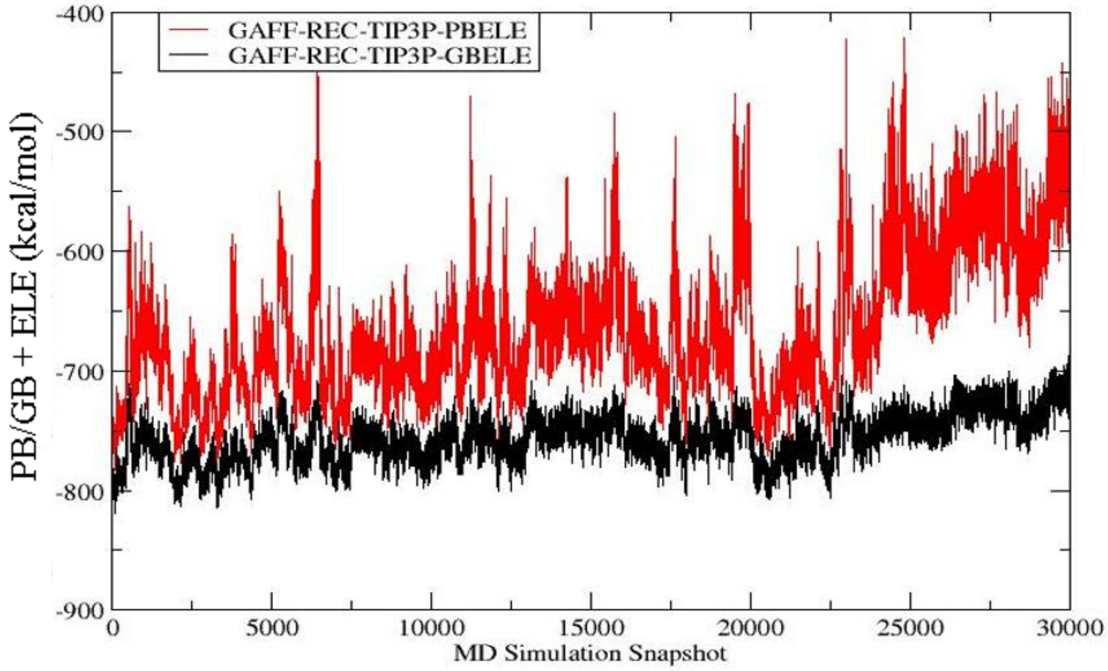

Table S1. Energy components of the three trajectories (host, guest and host-guest complex) under the GAFF parameter set, TIP3P solvation in sampling and PBSA implicit solvation in calculation.

Molecule	unbound						bound					
	H	±	TS	±	G	±	H	±	TS	±	G	±
WP6	-437.11	0.32	171.35	0.16	-608.46	0.36	-	-	-	-	-	-
G1	-39.56	0.02	34.03	0.03	-73.59	0.04	-548.18	0.10	177.58	0.17	-725.76	0.20
G2	-57.97	0.03	38.68	0.02	-96.65	0.03	-580.42	0.07	179.64	0.13	-760.06	0.15
G3	-10.10	0.02	24.54	0.00	-34.64	0.02	-516.60	0.11	171.09	0.12	-687.69	0.17
G4	-65.91	0.02	29.74	0.00	-95.65	0.02	-564.97	0.09	176.53	0.14	-741.50	0.17
G5	-40.18	0.03	42.04	0.00	-82.22	0.03	-546.13	0.13	183.41	0.13	-729.54	0.18
G6	-58.06	0.02	27.58	0.01	-85.64	0.02	-569.12	0.10	172.71	0.12	-741.83	0.16
G7	-77.14	0.02	24.60	0.01	-101.74	0.02	-575.18	0.12	171.03	0.11	-746.21	0.16
G8	-148.74	0.02	40.84	0.04	-189.58	0.05	-648.84	0.10	186.01	0.17	-834.85	0.20
G9	-381.96	0.02	29.00	0.03	-410.96	0.04	-907.81	0.09	171.25	0.10	-1079.06	0.13
G10	-187.48	0.02	41.11	0.00	-228.59	0.02	-702.14	0.08	182.45	0.20	-884.59	0.21
G11	-28.15	0.02	25.77	0.00	-53.92	0.02	-521.38	0.12	172.65	0.15	-694.03	0.19
G12	-137.16	0.03	40.64	0.00	-177.80	0.03	-667.13	0.07	178.74	0.11	-845.87	0.13
G13	47.22	0.02	35.36	0.00	11.86	0.02	-465.90	0.08	176.50	0.17	-642.40	0.19

Table S2. Energy components of the three trajectories (host, guest and host-guest complex) under the GAFF parameter set, TIP3P solvation in sampling and GBSA implicit solvation in calculation.

Molecule	unbound						bound					
	H	±	TS	±	G	±	H	±	TS	±	G	±
WP6	-528.82	0.11	171.35	0.16	-700.17	0.19	-	-	-	-	-	-
G1	-36.43	0.02	34.03	0.03	-70.46	0.04	-589.01	0.07	177.58	0.17	-766.59	0.19
G2	-57.72	0.03	38.68	0.02	-96.40	0.03	-619.80	0.06	179.64	0.13	-799.44	0.15
G3	-7.81	0.02	24.54	0.00	-32.35	0.02	-564.90	0.07	171.09	0.12	-735.99	0.14
G4	-69.44	0.02	29.74	0.00	-99.18	0.02	-611.42	0.07	176.53	0.14	-787.95	0.16
G5	-39.17	0.03	42.04	0.00	-81.21	0.03	-591.23	0.08	183.41	0.13	-774.64	0.15
G6	-55.47	0.02	27.58	0.01	-83.05	0.02	-612.79	0.07	172.71	0.12	-785.50	0.14
G7	-74.93	0.02	24.60	0.01	-99.53	0.02	-628.19	0.07	171.03	0.11	-799.22	0.13
G8	-146.27	0.02	40.84	0.04	-187.11	0.05	-686.19	0.07	186.01	0.17	-872.20	0.19
G9	-375.98	0.02	29.00	0.03	-404.98	0.04	-956.42	0.07	171.25	0.10	-1127.67	0.12
G10	-187.07	0.03	41.11	0.00	-228.18	0.03	-743.12	0.06	182.45	0.20	-925.57	0.21
G11	-26.94	0.02	25.77	0.00	-52.71	0.02	-573.54	0.07	172.65	0.15	-746.19	0.16
G12	-136.37	0.03	40.64	0.00	-177.01	0.03	-703.31	0.06	178.74	0.11	-882.05	0.13
G13	43.80	0.02	35.36	0.00	8.44	0.02	-515.74	0.07	176.50	0.17	-692.24	0.18

Table S3. Energy components of the three trajectories (host, guest and host-guest complex) under the GAFF parameter set, SPC/E solvation in sampling and PBSA implicit solvation in calculation.

Molecule	unbound						bound					
	H	±	TS	±	G	±	H	±	TS	±	G	±
WP6	-315.48	0.47	170.02	0.15	-485.50	0.49	-	-	-	-	-	-
G1	-39.07	0.02	34.12	0.02	-73.19	0.03	-564.45	0.07	175.49	0.08	-739.94	0.07
G2	-57.96	0.03	38.68	0.02	-96.64	0.03	-581.18	0.07	179.66	0.13	-760.84	0.07
G3	-10.12	0.02	24.54	0.00	-34.66	0.02	-517.97	0.11	171.59	0.13	-689.56	0.17
G4	-65.93	0.02	29.74	0.00	-95.67	0.02	-564.31	0.10	176.50	0.14	-740.81	0.12
G5	-40.20	0.03	42.04	0.00	-82.24	0.03	-557.55	0.07	184.15	0.09	-741.70	0.15
G6	-58.14	0.02	27.56	0.01	-85.70	0.02	-558.09	0.19	172.69	0.13	-730.78	0.23
G7	-77.32	0.02	24.52	0.01	-101.84	0.02	-582.77	0.10	171.95	0.16	-754.72	0.17
G8	-148.70	0.02	40.79	0.05	-189.49	0.05	-643.77	0.10	186.22	0.17	-829.99	0.13
G9	-381.96	0.02	29.00	0.04	-410.96	0.04	-908.82	0.07	171.49	0.10	-1080.31	0.15
G10	-187.50	0.02	41.11	0.00	-228.61	0.02	-702.01	0.08	182.10	0.18	-884.11	0.18
G11	-28.14	0.02	25.77	0.00	-53.91	0.02	-521.68	0.11	172.90	0.16	-694.58	0.21
G12	-137.17	0.03	40.64	0.00	-177.81	0.03	-668.06	0.07	179.05	0.11	-847.11	0.12
G13	47.23	0.02	35.36	0.00	11.87	0.02	-466.15	0.09	176.57	0.12	-642.72	0.20

Table S4. Energy components of the three trajectories (host, guest and host-guest complex) under the GAFF parameter set, SPC/E solvation in sampling and GBSA implicit solvation in calculation.

Molecule	unbound						bound					
	H	±	TS	±	G	±	H	±	TS	±	G	±
WP6	-498.46	0.11	170.02	0.15	-668.48	0.18	-	-	-	-	-	-
G1	-36.04	0.02	34.12	0.02	-70.16	0.03	-595.31	0.06	175.49	0.08	-770.80	0.10
G2	-57.71	0.03	38.68	0.02	-96.39	0.03	-620.59	0.06	179.66	0.13	-800.25	0.15
G3	-7.83	0.02	24.54	0.00	-32.37	0.02	-564.71	0.07	171.59	0.13	-736.30	0.14
G4	-69.46	0.02	29.74	0.00	-99.20	0.02	-611.38	0.07	176.50	0.14	-787.88	0.15
G5	-39.19	0.03	42.04	0.00	-81.23	0.03	-597.30	0.06	184.15	0.09	-781.45	0.11
G6	-55.55	0.02	27.56	0.01	-83.11	0.02	-608.06	0.08	172.69	0.13	-780.75	0.15
G7	-75.03	0.02	24.52	0.01	-99.55	0.02	-628.16	0.07	171.95	0.16	-800.11	0.17
G8	-146.22	0.02	40.79	0.05	-187.01	0.05	-682.62	0.08	186.22	0.17	-868.84	0.19
G9	-375.97	0.02	29.00	0.04	-404.97	0.04	-956.92	0.07	171.49	0.10	-1128.41	0.12
G10	-187.08	0.03	41.11	0.00	-228.19	0.03	-743.25	0.07	182.10	0.18	-925.35	0.19
G11	-26.93	0.02	25.77	0.00	-52.70	0.02	-572.00	0.07	172.90	0.16	-744.90	0.17
G12	-136.38	0.03	40.64	0.00	-177.02	0.03	-704.10	0.06	179.05	0.11	-883.15	0.13
G13	43.82	0.02	35.36	0.00	8.46	0.02	-516.40	0.07	176.57	0.12	-692.97	0.14

Table S5. Energy components of the three trajectories (host, guest and host-guest complex) under the GAFF2 parameter set, TIP3P solvation in sampling and PBSA implicit solvation in calculation.

Molecule	unbound						bound					
	H	±	TS	±	G	±	H	±	TS	±	G	±
WP6	-205.62	1.06	161.50	0.15	-367.12	1.07	-	-	-	-	-	-
G1	-39.56	0.02	34.03	0.03	-73.59	0.04	-558.09	0.07	176.54	0.12	-734.63	0.14
G2	-57.97	0.03	38.68	0.02	-96.65	0.03	-581.47	0.07	178.10	0.15	-759.57	0.16
G3	-10.10	0.02	24.54	0.00	-34.64	0.02	-512.13	0.09	171.29	0.14	-683.42	0.16
G4	-65.91	0.02	29.74	0.00	-95.65	0.02	-557.72	0.09	177.02	0.16	-734.74	0.18
G5	-40.18	0.03	42.04	0.00	-82.22	0.03	-558.95	0.07	183.58	0.10	-742.53	0.12
G6	-58.06	0.02	27.58	0.01	-85.64	0.02	-566.80	0.07	173.71	0.14	-740.51	0.16
G7	-77.14	0.02	24.60	0.01	-101.74	0.02	-573.69	0.10	172.01	0.17	-745.70	0.19
G8	-148.74	0.02	40.84	0.04	-189.58	0.05	-650.61	0.08	185.04	0.17	-835.65	0.19
G9	-381.96	0.02	29.00	0.03	-410.96	0.04	-894.89	0.07	172.88	0.11	-1067.77	0.13
G10	-187.48	0.02	41.11	0.00	-228.59	0.02	-703.64	0.07	180.94	0.21	-884.58	0.22
G11	-28.15	0.02	25.77	0.00	-53.92	0.02	-520.85	0.09	172.91	0.15	-693.76	0.17
G12	-137.16	0.03	40.64	0.00	-177.80	0.03	-664.90	0.06	178.22	0.12	-843.12	0.14
G13	47.22	0.02	35.36	0.00	11.86	0.02	-458.77	0.07	176.06	0.13	-634.83	0.15

Table S6. Energy components of the three trajectories (host, guest and host-guest complex) under the GAFF2 parameter set, TIP3P solvation in sampling and GBSA implicit solvation in calculation.

Molecule	unbound						bound					
	H	±	TS	±	G	±	H	±	TS	±	G	±
WP6	-448.63	0.20	161.50	0.15	-610.13	0.25	-	-	-	-	-	-
G1	-36.43	0.02	34.03	0.03	-70.46	0.04	-586.85	0.06	176.54	0.12	-763.39	0.14
G2	-57.72	0.03	38.68	0.02	-96.40	0.03	-618.03	0.06	178.10	0.15	-796.13	0.16
G3	-7.81	0.02	24.54	0.00	-32.35	0.02	-553.15	0.07	171.29	0.14	-724.44	0.15
G4	-69.44	0.02	29.74	0.00	-99.18	0.02	-600.53	0.07	177.02	0.16	-777.55	0.17
G5	-39.17	0.03	42.04	0.00	-81.21	0.03	-596.16	0.06	183.58	0.10	-779.74	0.12
G6	-55.47	0.02	27.58	0.01	-83.05	0.02	-602.76	0.06	173.71	0.14	-776.47	0.15
G7	-74.93	0.02	24.60	0.01	-99.53	0.02	-616.54	0.07	172.01	0.17	-788.55	0.18
G8	-146.27	0.02	40.84	0.04	-187.11	0.05	-682.07	0.07	185.04	0.17	-867.11	0.18
G9	-375.98	0.02	29.00	0.03	-404.98	0.04	-940.55	0.06	172.88	0.11	-1113.43	0.13
G10	-187.07	0.03	41.11	0.00	-228.18	0.03	-740.48	0.06	180.94	0.21	-921.42	0.22
G11	-26.94	0.02	25.77	0.00	-52.71	0.02	-563.94	0.06	172.91	0.15	-736.85	0.16
G12	-136.37	0.03	40.64	0.00	-177.01	0.03	-698.83	0.06	178.22	0.12	-877.05	0.14
G13	43.80	0.02	35.36	0.00	8.44	0.02	-504.08	0.06	176.06	0.13	-680.14	0.14

Table S7. Energy components of the three trajectories (host, guest and host-guest complex) under the GAFF2 parameter set, SPC/E solvation in sampling and PBSA implicit solvation in calculation.

Molecule	unbound						bound					
	H	±	TS	±	G	±	H	±	TS	±	G	±
WP6	-361.51	0.50	162.63	0.14	-524.14	0.52	-	-	-	-	-	-
G1	-39.07	0.02	34.12	0.02	-73.19	0.03	-552.06	0.09	176.52	0.14	-728.58	0.16
G2	-57.96	0.03	38.68	0.02	-96.64	0.03	-583.00	0.06	178.10	0.15	-761.10	0.16
G3	-10.12	0.02	24.54	0.00	-34.66	0.02	-514.62	0.09	171.45	0.15	-686.07	0.18
G4	-65.93	0.02	29.74	0.00	-95.67	0.02	-565.24	0.07	177.19	0.17	-742.43	0.18
G5	-40.20	0.03	42.04	0.00	-82.24	0.03	-560.75	0.07	183.57	0.09	-744.32	0.11
G6	-58.14	0.02	27.56	0.01	-85.70	0.02	-567.48	0.07	174.08	0.17	-741.56	0.18
G7	-77.32	0.02	24.52	0.01	-101.84	0.02	-576.39	0.09	171.85	0.20	-748.24	0.22
G8	-148.70	0.02	40.79	0.05	-189.49	0.05	-654.86	0.08	184.95	0.16	-839.81	0.18
G9	-381.96	0.02	29.00	0.04	-410.96	0.04	-893.34	0.08	172.96	0.11	-1066.30	0.14
G10	-187.50	0.02	41.11	0.00	-228.61	0.02	-706.86	0.07	180.18	0.19	-887.04	0.20
G11	-28.14	0.02	25.77	0.00	-53.91	0.02	-524.45	0.09	173.11	0.17	-697.56	0.19
G12	-137.17	0.03	40.64	0.00	-177.81	0.03	-666.82	0.06	177.91	0.12	-844.73	0.13
G13	47.23	0.02	35.36	0.00	11.87	0.02	-461.83	0.07	175.96	0.13	-637.79	0.15

Table S8. Energy components of the three trajectories (host, guest and host-guest complex) under the GAFF2 parameter set, SPC/E solvation in sampling and GBSA implicit solvation in calculation.

Molecule	unbound						bound					
	H	±	TS	±	G	±	H	±	TS	±	G	±
WP6	-474.51	0.14	162.63	0.14	-637.14	0.20	-	-	-	-	-	-
G1	-36.04	0.02	34.12	0.02	-70.16	0.03	-584.01	0.07	176.52	0.14	-760.53	0.15
G2	-57.71	0.03	38.68	0.02	-96.39	0.03	-619.49	0.06	178.10	0.15	-797.59	0.16
G3	-7.83	0.02	24.54	0.00	-32.37	0.02	-554.39	0.07	171.45	0.15	-725.84	0.16
G4	-69.46	0.02	29.74	0.00	-99.20	0.02	-604.12	0.06	177.19	0.17	-781.31	0.18
G5	-39.19	0.03	42.04	0.00	-81.23	0.03	-597.58	0.06	183.57	0.09	-781.15	0.11
G6	-55.55	0.02	27.56	0.01	-83.11	0.02	-602.67	0.06	174.08	0.17	-776.75	0.18
G7	-75.03	0.02	24.52	0.01	-99.55	0.02	-616.26	0.06	171.85	0.20	-788.11	0.21
G8	-146.22	0.02	40.79	0.05	-187.01	0.05	-684.77	0.07	184.95	0.16	-869.72	0.17
G9	-375.97	0.02	29.00	0.04	-404.97	0.04	-938.75	0.07	172.96	0.11	-1111.71	0.13
G10	-187.08	0.03	41.11	0.00	-228.19	0.03	-742.72	0.06	180.18	0.19	-922.90	0.20
G11	-26.93	0.02	25.77	0.00	-52.70	0.02	-565.24	0.06	173.11	0.17	-738.35	0.18
G12	-136.38	0.03	40.64	0.00	-177.02	0.03	-700.41	0.06	177.91	0.12	-878.32	0.13
G13	43.82	0.02	35.36	0.00	8.46	0.02	-506.55	0.06	175.96	0.13	-682.51	0.14
